# Sensitivity to permanent non-functionality in Goffin’s cockatoos (*Cacatua goffiniana*)

**DOI:** 10.1038/s41598-026-57007-1

**Published:** 2026-06-09

**Authors:** Antonio J. Osuna-Mascaró, Eleonora Rovegno, Susana Monsó, Remco Folkertsma, Alice M. I. Auersperg

**Affiliations:** 1 Animal Innovation Research, Messerli Research Institute, University of Veterinary Medicine Vienna, Medical University Vienna, University of Vienna, Veterinaerplatz 1, Vienna, 1210 Austria; 2 Department of Life Sciences and Biotechnology, University of Ferrara, via Borsari 46, Ferrara, 44121 Italy; 3 Department of Logic, History and Philosophy of Science, UNED (Universidad Nacional de Educación a Distancia), Paseo Senda del Rey 7, Madrid, 28040 Spain; 4 Ethics and Human-Animal Studies, Messerli Research Institute, University of Veterinary Medicine Vienna, Medical University Vienna, University of Vienna, Veterinaerplatz 1, Vienna, 1210 Austria

**Keywords:** Non-functionality, Goffin’s cockatoos, Avian cognition, Tool use, Comparative thanatology, Animal behaviour, Psychology

## Abstract

While the ability to recognize that something is irreparably broken is important for human physical and social cognition, its underpinnings remain largely unexplored in non-human animals. Recognizing the permanent loss of function is highly relevant to foraging on perishable resources and to interacting with breakable objects such as wooden tools. Nevertheless, learning how to respond to such permanent non-functionality has broader implications, as it has been proposed to represent an important prerequisite for the development of a minimal concept of death. The Goffin’s cockatoo, an opportunistic extractive forager and skilled tool user both in the field and in captivity, has become a significant non-primate model for technical cognition and problem solving. Here, we investigate whether Goffin’s cockatoos can develop sensitivity to perishable touchscreen icons. In this set-up, the parrots flexibly and spontaneously categorized specific interactive elements as persistently non-functional after associating them with a single negative event.

**Supplementary Information:**

The online version contains supplementary material available at 10.1038/s41598-026-57007-1.

## Introduction

The ability to discern when a system has ceased to function and that this functional cessation is not temporally restricted is a fundamental aspect of human cognitive processing, particularly in the context of technology and social interactions. The recognition of irreversible non-functionality can be crucial for flexibly respond to changing environments, such as ceased resources, broken instruments, or departed people.

Learning to recognize and adapt to permanent non-functionality begins early in human development, starting with the most obvious functions (the lack of response expectation from dead individuals emerges around the age of three to five) and subsequently extending to more subtle ones (e.g., not expecting cognitive functions from deceased individuals has been documented among five- to eight-year-old children)^1,2^. This ability continues to be cultivated throughout adulthood and serves as an indispensable trait for a wide variety of actions, ranging from the mundane to complex and scientific endeavours (from broken toys to crops and to rocket science). It is important to note that permanent is understood in our research as defined by the Cambridge Dictionary as “lasting for a long time or forever,” and we use the term to refer to lasting for a long time.

Despite its significance to humans, the degree to which non-human animals understand permanent non-functionality has been largely overlooked in the scientific literature.

However, the capacity to flexibly recognize when something has irreversibly ceased to function may prove a highly valuable ability for non-human animals in a wide range of scenarios. For example, juvenile kea are granted a childhood-like phase during which they can displace any adult birds from resources and are sporadically fed by related and even unrelated male group members. Once they lose their yellow rings around the eyes, they will no longer be perceived as infants by the rest of the flock, and must learn that certain behaviours will no longer be tolerated in the same way^3^. A South African lioness would benefit from the ability to recognize when to release her throat clamp on her prey, preventing the animal from escaping. This is something that a solitary predator would likely learn after repeated exposure to the same situation^4,5^. Similarly, a Congo’s termite-fishing chimpanzee would save valuable time by recognizing that a previously strong stick has been splintered and is now useless for opening a hole in the termite mound to fish termites^6^.

Tool use provides most examples of studies focusing on the functionality of external objects, especially in our closest relatives: chimpanzees (*Pan troglodytes*). Being sensitive to the functionality of a tool not only allows for a flexible selection of a tool for a particular task, but it’s an important precondition to optimize tool behaviour. Chimpanzees have shown some sensitivity to tool functionality, being able to determine the suitability of tools for a task based on their functional properties rather than just their appearance^7,8^, and making, modifying and discarding tools depending on their current functionality for a particular task^9–11^.

Moreover, the relevance extends beyond tool use. The capacity to detect when a previously functional process has ceased to operate (and to treat that cessation as enduring) is emerging as a relevant topic within evolutionary thanatology, which examines how humans and other animals perceive and respond to death from an evolutionary perspective. Notably, it has been suggested that this ability is a prerequisite for acquiring a minimal concept of death, through the recognition of the permanent cessation of an organism’s externally perceptible functions^12^. This minimal form of death recognition has been argued to be potentially and relatively widespread^5^ as many animals share the ability to identify animacy and its violation^13–15^, and can learn cross-modally, resulting in implicit expectations of others’ behaviour^16–18^.

We tested Goffin’s cockatoos (*Cacatua goffiniana;* hereafter ‘Goffins’), an important non-primate model for technical cognition and tool use in a set-up targeting flexible sensitivity to permanent non-functionality. While Goffins lack the predispositions previously suggested to facilitate the emergence of tool use (nest building, food caching)^19,20^, they can spontaneously innovate highly complex modes of tool use and manufacture tools in both captive and wild settings^21–24^.

Notably, previous studies suggest that Goffins have a sensitivity to functionality in specific settings, acting flexibly to functional changes in the blocking elements in complex sequential problem-solving task^25^. Moreover, like chimpanzees, they have been reported to select, modify, and discard tools based on their contextual functionality^21,23,26^. They do not even attempt to employ a recently constructed tool before discarding it if they recognize it to be non-functional^27^.

Based on previous suggestive side findings we aimed to systematically test whether Goffins can express sensitivity to permanent non-functionality in novel situations and to what extent such sensitivity is flexibly expressed. Our experiment was designed with the objective of facilitating robust data collection while not relying on the use of tools to enable future comparisons.

We thus designed an experiment using a touchscreen interface. Comparative cognition has adapted the use of touchscreens to a wide diversity of animal models, including bumblebees, fish, dogs, and dolphins^28–31^, thus potentially allowing for the adaptation of this experimental design to other, distantly related species. In addition, this approach permits the set-up to be kept abstract, thereby avoiding interference with physical problem-solving experiences.

**Fig. 1:**
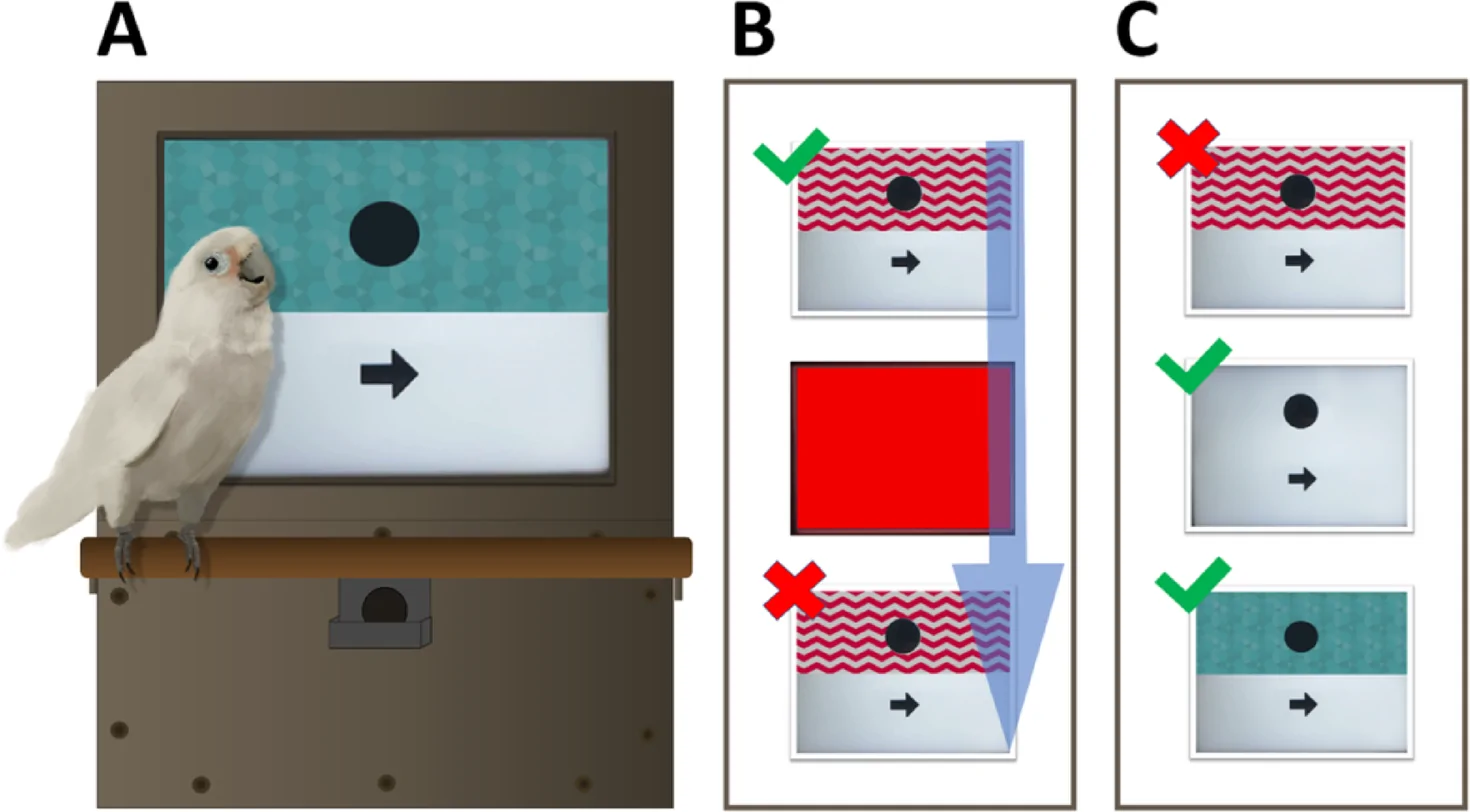
Apparatus and set-up. (**A**) Touchscreen apparatus with perch, reward chamber, and cockatoo. (B) Trials immediately before and after a flashing event; after the flashing event (centre, in red) the previous functional background (top) becomes non-functional (below). (**C**) After the change in functionality the background associated with the flashing event will remain non-functional (top), but controls will always be functional (centre), and the alternative background (below) will be functional until the same thing happens to it.

We tested fifteen Goffin’s cockatoos in a touchscreen task designed to assess sensitivity to persistent, background-specific loss of function. The birds were trained to interact with the touchscreen, where they encountered two different buttons: a round button on the top half of the screen with a rewarding function and an arrow button on the lower half of the screen for skipping to the next screen (Fig. 1A). During the testing phase, the subjects were presented with forty-two pseudorandomized trials per session. Of these trials, thirty-two had one of two differently coloured/patterned backgrounds on the top half of the screen (behind the round rewarding button), while the remaining ten were interspersed controls, in which both buttons were presented with a white background. Throughout the course of the experiment, the subjects received one hundred distinct backgrounds, which were presented in pairs for a total of fifty sessions.

During each session, a subject was expected to press the round button on every trial to receive a small nut reward. This continued until, on a given pseudorandomized trial, touching the round rewarding button was associated with a flashing event of the coloured background that rendered that button non-functional for the rest of the session (but with the flashing event only happening once per background). Moreover, it is crucial to note that the round button will only be non-functional when it is displayed against the same background that was present when the flashing event occurred. Thus, it will still be functional in trials with other backgrounds co-occurring in the same session.

We expected that, after a flashing event, subjects recognizing the same background as non-functional would be more likely to directly touch the arrow button to skip it, without first attempting to touch the round button. Because each session contained a new pair of backgrounds, improvement from one session to another would indicate that Goffins were transferring this relation between the flashing event, the background cue, and subsequent non-functionality to novel backgrounds. If they were able to identify functional backgrounds while spontaneously skipping non-functional ones across sessions, it can be inferred that they had acquired flexible sensitivity to permanent non-functionality.

## Results

All fifteen Goffins demonstrated engagement with the touchscreen in the test. Qualitatively, some individuals exhibited levels of arousal in response to the screen flashing and the button becoming non-functional (including, for example, vocalizations and crest raises). Notably, the two youngest subjects exhibited contrasting responses. Rose (female) displayed begging calls, typically used to obtain food or attention, while Renki (male) showed strong aggressive responses.

Some data were lost due to a malfunction in communication with the server, resulting in 39/50 sessions being recorded for one subject (Pipin), 44/50 for two subjects (Figaro and Irene), yet 45/50 or more for all the other Goffins.

### Overview of statistical models

We analysed the dataset using a planned set of nine mixed-effects models, grouped into four conceptual blocks. This structure was chosen because different parts of the task addressed distinct behavioural questions and, in some cases, required different response variables. Models A–D analysed first-choice accuracy in functional trials, non-functional trials, control trials, and flash-transition trials, respectively. First-choice accuracy was coded as a binomial response indicating whether the first button selected in a trial was correct. The correct response depended on the functional status of the trial: the round button was correct in functional and control trials, whereas the arrow button was correct in non-functional trials. In flash-transition trials, the correct response was the round button immediately before the flash and the arrow button immediately after the flash.

Model E analysed persistence, defined as the number of repeated presses on the now non-functional round button within a trial before the subject switched to the arrow. Model F analysed first-choice accuracy in unequal-status trials, in which one coloured background was still functional whereas the other had already become non-functional. Finally, Models G–I were complementary within-session background comparison analyses testing whether performance differed depending on whether a background was the first or second to become non-functional within a session. Model G used a count response similar to Model E, whereas Models H and I used first-choice accuracy on the first and second non-functional encounters of each background, respectively.

We used separate subsets because the behavioural demands were not equivalent across trial types. In functional and control trials, the correct response was the round button, whereas in non-functional trials the correct response was the arrow button. Flash-transition trials specifically tested the immediate change in response before versus after the flashing event, and persistence on non-functional trials was a count response rather than a binary correct/incorrect response. Thus, rather than fitting a single omnibus model combining all trial types, we used targeted models to test predefined behavioural contrasts while avoiding the conflation of functionally different trial contexts. Full details on error structures, random effects, scaling, model reduction, and diagnostics are provided in the Supplementary Information.

### Model A (functional trials)

This model examined only functional trials, i.e. trials where the round button was still rewarding. The model included session number, trial number, and their interaction as test predictors. The full–null model comparison showed a clear overall effect of these predictors (*χ*^2^ = 16.917, *df* = 1, *p* < 0.001). The model revealed a clearly significant interaction between session number and trial number (χ2 = 13.301, df = 1, *p* < 0.001). The direction of the effect suggests that individuals made some errors at the beginning of a session but soon improved from trial to trial, while by the end of the experiment they often pressed the correct button already at the beginning of a session and made slightly more errors in later trials (Fig. S2).

### Model B (non-functional trials)

**Fig. 2:**
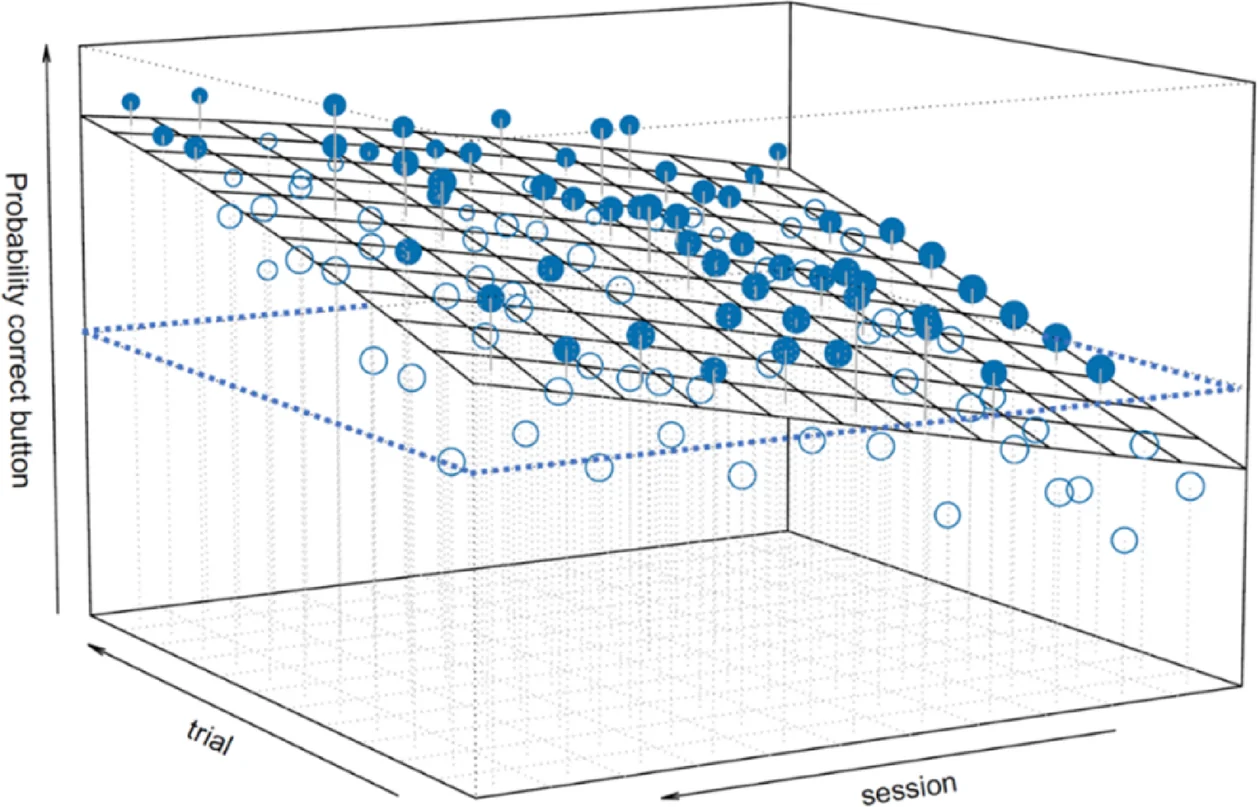
Non-functional trials (Model B). Probability of pressing the correct button when the screen is non-functional across trials and sessions. The gridded plane represents the group average. Dark bubbles are above the plane; white bubbles are below the plane. The blue horizontal line represents the 50–50 level. The size of the bubbles represents the distance from the viewer.

This model included only non-functional trials, i.e. reappearances of a background after its flash. The model included session number, trial number, and their interaction as test predictors. The full–null comparison again showed a clear overall effect of the predictors (χ2 = 31.197, df = 1, *p* < 0.001). However, the comparison between the full model and the respective reduced model showed that the interaction did not reach significance (χ2 = 0.082, df = 1, *p* = 0.774). When the interaction was removed, we found a clear positive effect of the number of trials (χ2 = 26.933, df = 1, *p* < 0.001), as well as a positive effect of the number of sessions (χ2 = 18.133, df = 1, *p* < 0.001) on the response, i.e. individuals made many errors in the beginning, but improved with increasing experience until the end of the experiment (Fig. 2).

### Model C (control trials)

This model included only control trials (white background, always functional). Test predictors were session number, trial number, number of non-functional screens, and all two-way and three-way interactions among them as test predictors. The full–null comparison revealed a significant overall effect of these predictors (χ2 = 59.016, df = 11, *p* < 0.001). However, the three-way interaction between session number, trial number, and number of non-functional screens was not significant (χ2 = 0.388, df = 2, *p* = 0.82), and none of the two-way interactions were significant. The final model containing only the main effects showed a significant effect of session number (χ2 = 23.162, df = 2, *p* < 0.001) as well as the number of non-functional screens (χ2 = 29.639, df = 1, *p* < 0.001), meaning that individuals’ performance improved with increasing exposure over time, but that their performance decreased as more screens became non-functional within each session (Fig. S3).

### Model D (flash-transition trials)

**Fig. 3:**
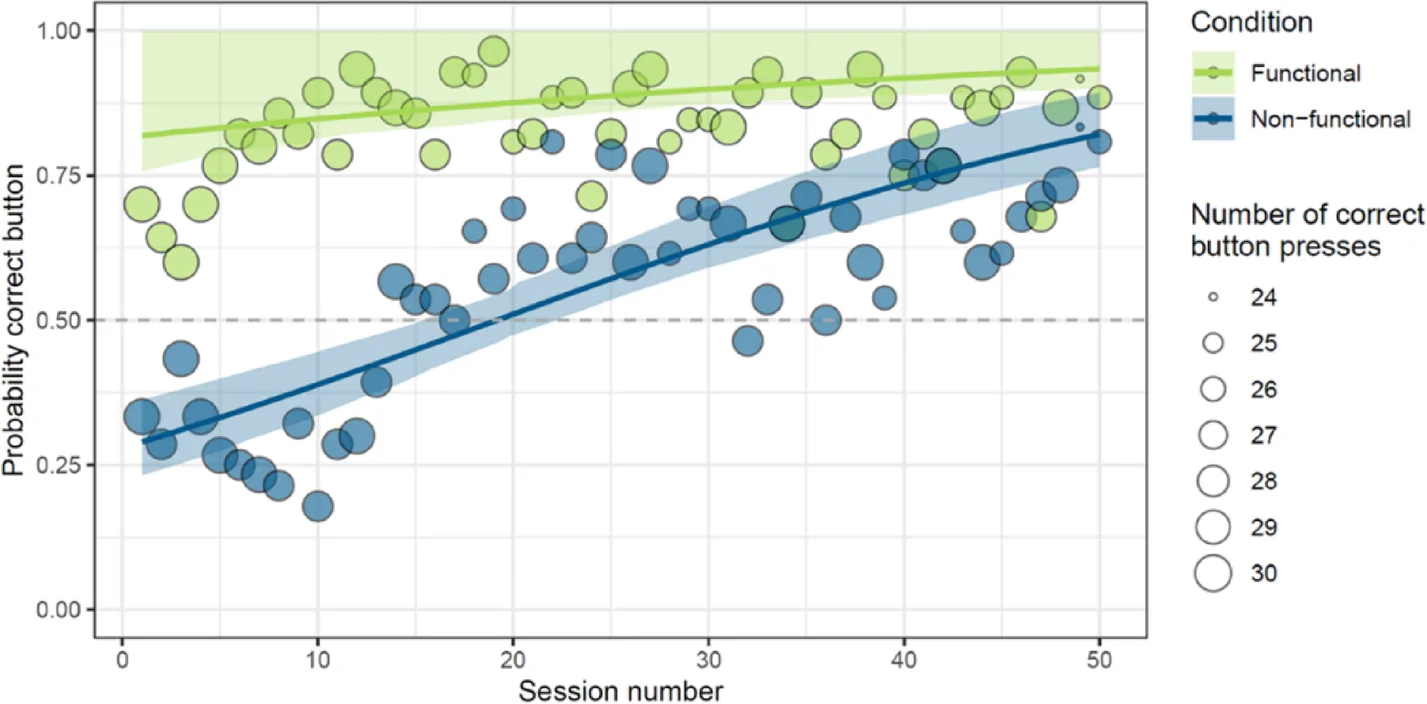
Before and after flashing events (Model D). Probability of choosing correctly immediately before and after changes in functionality. Colour represents the performance when facing functional (green) and non-functional (blue) trials. As the flashing event happened in a pseudorandomized fashion, the size of the bubbles represents the number of total button-presses when choosing.

This model targeted the two trials surrounding each flashing event. In these trials, the background is initially functional and becomes non-functional upon button press, requiring a different response. The model included background status, session number, and their interaction as test predictors. The full–null comparison revealed a significant overall effect of these predictors (χ² = 43.912, df = 3, *p* < 0.001). In particular, there was a significant interaction between background status and session number (χ² = 7.365, df = 1, *p* < 0.01), indicating that while the probability of choosing the correct button during functional trials increased only slightly across the experiment, performance increased sharply in the non-functional trial immediately following the flash transition (Fig. 3).

### Model E (persistence on non-functional trials)

This model quantified how many times subjects repeatedly pressed the now non-functional round button within a single non-functional trial. This model included trial number, session number, and their interaction as test predictors. The full–null comparison supported the predictors as a set (χ2 = 41.440, df = 1, *p* < 0.001) of the test predictors. The session number × trial number interaction was also supported (χ2 = 4.256, df = 1, *p* < 0.039). At the beginning of earlier sessions, individuals continued to press the non-functional round button frequently before moving on to the arrow button, but did so less over trials, whereas at the beginning of later sessions, individuals pressed the non-functional button less often and showed a smaller decrease over trials (Fig. S4).

### Model F (unequal-status trials)

This model tests whether subjects can selectively adjust behaviour when one background changes status while the alternative background remains functional. Again, the full-null model comparison revealed an effect of the test predictors (see ESM for details) as a whole. The model included background status, session number, and their interaction as test predictors. The full–null comparison showed a significant overall effect of these predictors (χ2 = 34.544, df = 3, *p* < 0.001), just as in Model D, we found a significant interaction between status and session number (χ2 = 7.156, df = 1, *p* < 0.01). The model predicted that while the probability of pressing the correct button across trials did not change for functional screens, individuals’ probability of pressing the correct button increased significantly for non-functional trials (Fig. S5).

### Model G: incorrect presses after functionality loss

Model G was conceptually related to Model E but addressed a narrower within-session question. Whereas Model E tested how persistence on non-functional trials changed overall across sessions and trials, Model G tested whether this persistence differed depending on whether a background was the first or second to become non-functional within a session. We therefore analysed how often birds continued to press the non-functional round button after the flashing event. The model included background number (first vs. second), session number, and trial number as test predictors, together with their three-way interaction. Sex and the status of the other background were included as control predictors.

The full–null model comparison revealed a significant overall effect of the test predictors (χ² = 59.176, df = 7, *p* < 0.001). After model reduction, an almost significant interaction between session number and trial number remained (χ² = 3.358, df = 1, *p* = 0.059), but we found no evidence that background order affected persistence (χ² = 0.078, df = 1, *p* = 0.779). Thus, birds did not differ in their within-trial persistence depending on whether the background was the first or second to become non-functional within a session.

### Model H: probability of correct responding on the first encounter

We next examined the first non-functional encounter for each background. The model included background number, session number, their interaction, and sex (Fig. S6).

The full–null comparison indicated significant effects overall (χ² = 24.159, df = 3, *p* < 0.001), but the background number × session number interaction was non-significant (χ² = 2.669, df = 1, *p* = 0.103). After model reduction, background number showed no effect (χ² = 0.397, df = 1, *p* = 0.528), whereas session number showed a strong positive effect (χ² = 21.477, df = 1, *p* < 0.001). Performance was very similar for the two backgrounds (background 1: P(correct) = 0.548 ± 0.036; background 2: 0.572 ± 0.026), and improved robustly across sessions (β = 0.677).

### Model I: probability of correct responding on the second encounter

Finally, we analysed the second encounter with each background—i.e., the first time that birds needed to classify the background as non-functional *without* the flash cue (Fig. S6).

The full–null comparison again revealed significant effects overall (χ² = 23.052, df = 3, *p* < 0.001), without a significant interaction (χ² = 0.006, df = 1, *p* = 0.941). After model reduction, we found significant effects of both background number (χ² = 22.121, df = 1, *p* < 0.001) and session number (χ² = 5.207, df = 1, *p* = 0.023). Birds were more accurate on the second background during this more demanding discrimination step (background 1: P(correct) = 0.323 ± 0.036; background 2: 0.498 ± 0.041), and performance again increased across sessions (β = 0.181).

### Performance of first and last sessions compared

**Fig. 4:**
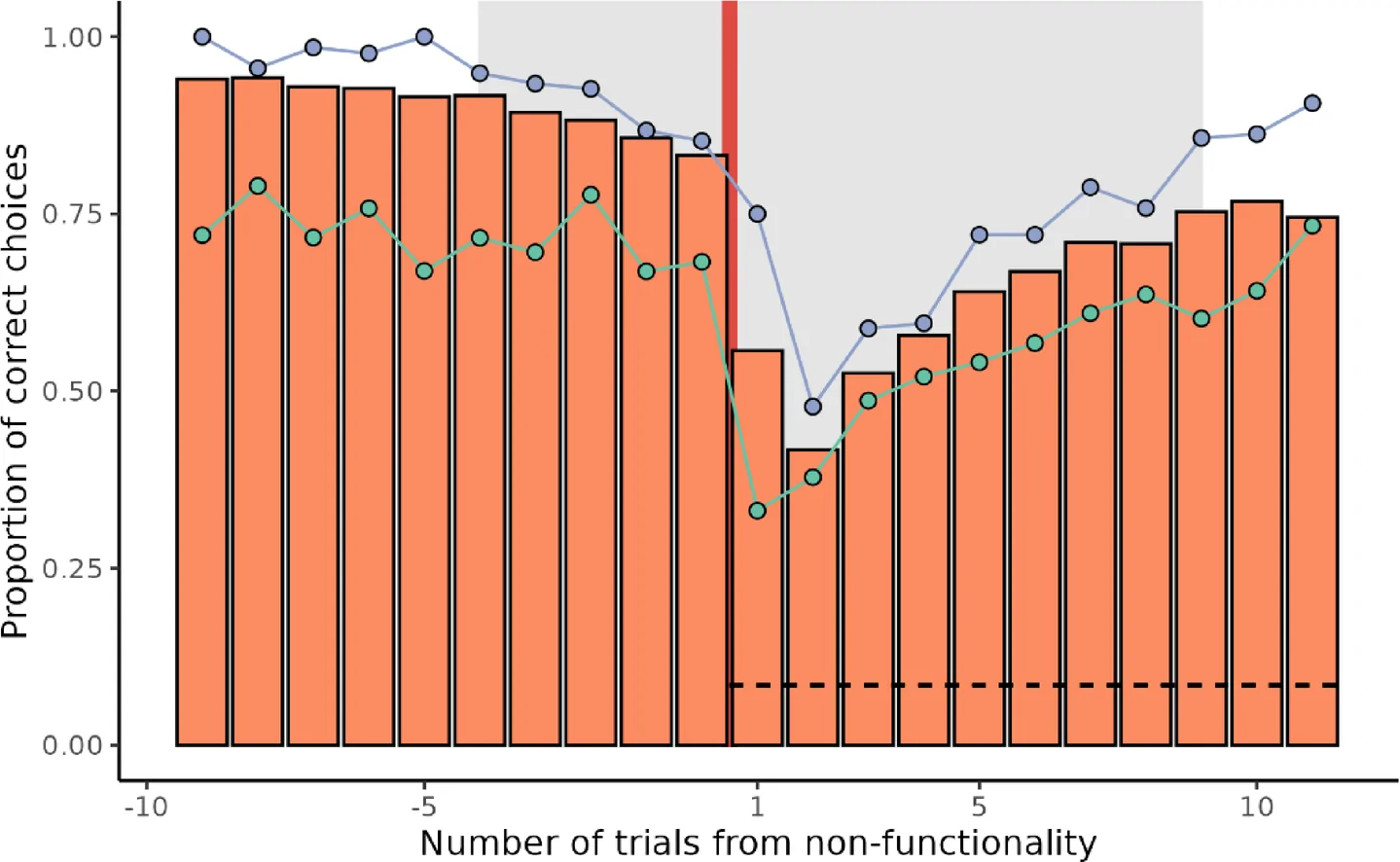
First and last five sessions compared. This graph shows the average and compressed performance for both backgrounds near the change in functionality over the entire experiment and a comparison between the first and last five sessions (first sessions in green, last sessions in blue). The red column represents the change in functionality, all trials on the left are functional and all trials on the right are non-functional. The orange columns represent the average performance along the trial. The horizontal dotted line represents the expected performance on non-functional trials if they didn’t change their behaviour after the change in functionality (H_0_). The grey area represents the trials in which the change in functionality can happen. The dip on the second encounter reflects the attentional shift required to identify the background as non-functional without the accompanying flash.

To illustrate how performance changed around the transition from functional to non-functional trials, we compared the response profile in the first and last five sessions of the experiment. This descriptive analysis complements the model-based results by showing whether birds’ behaviour around the flashing event changed with experience, and whether they became more likely to select the arrow button on later encounters with a background that had become non-functional. We visualised this pattern both at the group level (Fig. 4) and for each subject individually (Fig. S7).

This comparison revealed several noteworthy trends. First, there was a general improvement in overall performance, as evidenced also by Model A. Second, there was a decline in performance when encountering the first non-functional trial, which was initially strong but weakened towards the end of the experiment. Third, performance typically dipped on the first non-flash reappearance of a newly non-functional background. This is expected because, unlike the flash trial, this reappearance provided no salient cue and required relying solely on the background pattern. Although performance declined relative to the flash trial, it remained far above the null prediction (0.085), and recovered over further re-encounters.

When their performance was analysed individually (as shown in Fig. S7), strong differences between individual performances were observed. Almost all individuals had excellent performance on functional trials during the final sessions of the experiment, with some showing a greater change in non-functional trials than others. Importantly, during the last sessions, some subjects showed no decline in performance during the first or even the second trial with a non-functional background. In the last five sessions, no subject’s performance on the first encounter with non-functional trials (i.e., first non-functional trial) fell below the 0.5 two-choice probability benchmark, and some didn’t change or even improved their performance during this encounter (see DOL, DOR, HEI, IRE, PIP, and MUP in Fig. S7). The second encounter with a non-functional background had a stronger effect on performance for most of them (with some individuals falling below chance), but a few had excellent performance or improved with respect to the first encounter (see DOR, JAN, and KIW in Fig. S7). Representing the first and last five sessions, the aforementioned data loss had a noticeable impact on the graphs of a few subjects (e.g., PIP & FIN).

To quantify whether performance in the first non-functional trials exceeded what would be expected if birds had not adjusted their responses after functionality loss, we compared performance in the first three non-functional encounters with a baseline derived from the fifth test trial within each session. This was the last trial position at which both coloured backgrounds were still guaranteed to be functional. Birds selected the correct round button in 91.5% of these fifth-trial baseline trials; therefore, if they had continued to select the round button after functionality loss, the expected rate of correct arrow-button responses in non-functional trials would be 1 − 0.915 = 0.085. Performance in the first three non-functional encounters was significantly above this expectation in both the first and last five sessions (Table S1; Supplementary Information).

## Discussion

Over the course of our study, Goffins became increasingly proficient at recognizing backgrounds whose associated button had become permanently non-functional within the session. Birds rapidly adjusted on flash trials and, with experience, also learned to classify backgrounds as non-functional on later reappearances without the flash. Their performance on these later reappearances (requiring an attentional shift from the salient flash to the background cue) improved markedly within and across sessions.

Control-trial performance remained high throughout, demonstrating that the presence of non-functional backgrounds did not suppress round-button use more broadly. The pattern across Models A–F shows that subjects learned background-specific, persistent mappings: once a background had yielded a flash and a non-reinforced outcome, they increasingly treated it as non-functional while maintaining appropriate responses to other backgrounds. This is in line with previous observations of Goffins seemingly recognizing non-functionality in previously functional tools and locking devices during problem solving^25,27^, and optimizing their actions accordingly when this implies an extra investment of energy and time^26^ and a certain degree of impulse control^25,32^.

Unsurprisingly, as they had learned in the training, they kept choosing the round buttons in functional trials at high rates. Nevertheless, they also improved their ability to choose the arrow button over the non-functional round button within and between sessions over time. This indicates that they learned to successfully transfer the function of the arrow from the training to the testing situation. This interpretation is further supported by the within-session background comparison analyses (Models G–I), which showed that birds performed similarly on the two backgrounds at their first non-functional encounter, but were significantly more accurate on the second background at the more demanding second encounter, with performance improving reliably across sessions.

To avoid a loss of general motivation throughout the experiment due to both backgrounds becoming non-functional, we introduced a series of pseudorandomized white-background control trials that were not subject to any alterations in functionality as a result of the test trials. The performance of the animals with these controls remained comparable to their performance when encountering functional trials. While the number of non-functional trials already present during the session had a modest influence on the controls’ performance, even in the most unfavourable circumstances (i.e., when a control was encountered when both backgrounds were non-functional and during the initial sessions of the experiment), their performance was high.

The Goffins did not generalize non-functionality to the controls, which indicates that they associated the functionality with the backgrounds behind the round button in the test trials.

A simple win-stay/lose-shift strategy is unlikely to fully explain the observed pattern. Such a strategy would predict a local shift away from the round button after an unrewarded response, but it would not by itself predict background-specific responding across later reappearances. In our task, the round button remained rewarded in control trials and in the alternative coloured background until that background also flashed. Thus, after a loss on one background, a simple lose-shift response would risk producing inappropriate arrow choices on still-functional backgrounds. Instead, birds increasingly skipped the specific background that had become non-functional while continuing to press the round button in functional and control trials. This pattern is more consistent with learning a background-specific change in functionality than with a general shift away from the round button.

To further assess this interpretation, we examined birds’ first-choice responses around the transition from functionality to non-functionality. They performed well on functional trials throughout the experiment, but increasingly selected the arrow button after a flashing event and on later reappearances of the same non-functional background.

This interpretation is further supported by the finding that performance in the first non-functional trials was significantly higher than the conservative null expectation derived from pre-transition functional trials. Thus, birds did not simply continue to apply the previously rewarded round-button response after functionality loss. Moreover, across the experiment, they increasingly selected the arrow button immediately after the first flashing event of a background, reaching selection rates in non-functional trials that were comparable to those in functional trials by the end of the study.

The combined performance of subjects in functional and non-functional trials, both before and after the flashing event, constitutes evidence of their capacity to distinguish between backgrounds, recognize them, and identify them with regard to their functionality within event trials.

We show that Goffins improve their general performance on non-functional trials over the course of the experiment and that they could clearly learn to associate the flashing event with the immediate non-functionality of the button. However, to test the extent to which the birds learned about the persistence of non-functionality of a particular slide beyond a within trial association with the flashing event, required additionally investigating their spontaneous (first icon touched) performance on the subsequent trials in which the same background reappeared (now again without the flashing event but still non-functional).

By the end of the experiment, the gap in performance between the first encounter with a non-functional background and later encounters diminished, with birds eventually achieving arrow-button selections at rates similar to their round-button selections in functional trials. In contrast, if the birds had only learned the flashing event without generalizing the non-functionality of the background, their responses would have remained close to the null prediction.

In our setup, the onset of non-functionality was marked by two sequential cues: an intensely salient flashing event and, on every later appearance, the background pattern that had been present during that flash. Because the flash never reappeared, subjects had to redirect attention from the conspicuous, formerly informative cue to a stimulus that had previously been incidental, a classic attentional-shift challenge^33^. This demand was evident when accuracy dipped the first time the birds re-encountered the same background in the absence of the flash; nevertheless, they rapidly adapted, switching to the arrow button on subsequent trials and thereby demonstrating that they had learned the background-specific, persistent non-functionality.

At the beginning of a session, the birds mainly faced trials in which the button was functional for many consecutive trials. Thus, when a flashing event happens, in a correct trial, we expect the bird’s attention to be focused on the button before the event (still during a functional trial), on the highly salient flash during the event, and on the arrow after the event (already during a non-functional trial). This could distract from the background of the trial, making it difficult to immediately achieve high performance on the very next trial. This second exposure to a non-functional background is a possible explanation for the observed decline in performance in our analysis of unequal conditions relative to the performance immediately before and after the change in functionality. Nevertheless, their performance on the second and subsequent encounters with a non-functional trial was well above what would be expected from the null hypothesis.

Moreover, the comparison of the first and last five sessions of the entire experiment revealed that some individuals exhibited better performances on the second exposure to the non-functional background than on the first. This may well be attributed to differences in impulse control, and arguably, to the ability to attend to the background when the change in functionality occurred. Although our observations hint at possible sex- or age-related differences in performance (with young female subjects performing better), our limited sample size does not permit definitive conclusions. Thus, these preliminary trends should be interpreted with caution and considered primarily as hypotheses to be tested in future studies with larger sample sizes.

Overall, most Goffins improved markedly across sessions and, at the group level, performed very well on non-functional trials (Fig. 2). Nevertheless, their individual performance varied considerably; factors such as age, sex, or personality—variables not controlled for in the present study—likely contributed to these differences.

These parrots demonstrated the ability to recognize functional backgrounds while improving their ability to skip non-functional backgrounds from one session to another, and did not generalize the change in functionality to control trials. This suggests that they are able to generalize the rule by acquiring the required flexible sensitivity to permanent non-functionality.

Whether this experimental design can be used to argue that Goffins meet the basic cognitive requirements to develop a minimal concept of death depends on their perception of traits that can be noticed as functions in living beings^5,12^. From our perspective, expecting a particular behaviour from an object, or as in this case an interactive touchscreen icon, may in its essence, not be drastically different from having those expectations from a living being. Indeed, for a social animal with sophisticated cognition, the latter may even be easier to learn^15^. However, this is open to debate and deviates from the objectives of this study.

A limitation of the present study is that it cannot determine whether Goffins represent permanent non-functionality in a human-like conceptual sense. The task demonstrates flexible sensitivity to a persistent loss of function within the experimental context, but the observed behaviour could still be supported by associative learning, operant conditioning, and attention to background-specific reinforcement histories. For this reason, we interpret the findings as evidence for sensitivity to persistent, background-specific non-functionality rather than as evidence that the birds fully understand irreversibility or permanent loss of function in an abstract sense. Future studies could test the limits of this sensitivity by introducing longer delays, novel transfer contexts, different modalities, or cases in which functionality cannot be restored across longer timescales.

The present study provides evidence that Goffins can learn about non-functionality and respond flexibly to it, and it is consistent with previous side findings^23,25,26^. This ability may prove to be highly valuable to species that instrumentally use perishable objects and are able to adapt flexibly to the outcomes of their use. However, it is conceivable that this could prove beneficial to a wide range of other species, including, for example, predators and nest builders.

Our experimental set-up has the potential to be employed in a wide variety of animal models. However, comparisons between species will need to be evaluated in accordance with the potential modifications made (e.g., by including time penalties).

## Materials and methods

### Subjects

Fifteen Goffins (including thirteen adults, six females and seven males, hatched between 2007 and 2011; and two juveniles, a male and a female, hatched in 2020 and 2021, respectively) participated in this experiment. All the Goffin Lab’s birds are captive-born and purchased from European breeders who are accredited and have full CITES certificates. They are officially registered at the district’s administrative animal welfare bureau in accordance with the Austrian Animal Protection Act § 25—TschG. BGBl. 118. The birds are permanently housed under environmentally and socially enriched conditions at the Goffin Lab in Goldegg (Lower Austria), with an indoor aviary measuring 45 m^2^ and an outdoor aviary measuring approximately 200 m^2^. The indoor aviary is 3–6 m high, while the outdoor aviary is 3–4.5 m high. The birds are provided with a diet that is varied and predominantly fresh and are never food-deprived (special treats such as nuts are reserved for experimental procedures). The group is kept at a minimum temperature of 17 °C during the winter, with a 12:12 light and dark cycle. With the exception of the two juvenile birds, all the subjects had prior experience with tools^21,23^ and with touchscreens^34^.

### Ethics

This study involved strictly non-invasive, voluntary interaction with a touchscreen device, and did not qualify as an animal experiment under the EU Directive 2010/63/EU and §§ 2–3 of the Austrian Tierversuchsgesetz 2012 (TVG 2012, BGBl. I 114/2012, idF BGBl. I 76/2020). All subjects were captive-bred in Europe (CITES-compliant) and are registered with the local Animal Welfare Bureau (Bezirkshauptmannschaft St. Pölten, NÖ). The birds are free-flying (no wing clipping) and permanently housed in socially and environmentally enriched aviaries, including an indoor aviary (45 m², 3–6 m high) and an outdoor aviary (150 m², 3–4.5 m high). Fresh fruits, seeds, protein sources, minerals, and water were available ad libitum. Housing conditions comply with the Austrian Animal Protection Act (§ 24 Abs. 1 Z 1 and 2; § 25 Abs. 3 TSchG). Participation in the study was entirely voluntary: individuals were called by name and entered the testing room only if they chose to. This study adheres to the ARRIVE 2.0 guidelines, and all relevant checklist items (animal details, housing, enrichment, welfare monitoring, study design, and statistical analysis) are fully reported in the Methods section.

### Apparatus

The touchscreen device (Fig. 1A) was an adapted mobile version of the Vienna Comparative Cognition Technology, an operant-conditioning system developed for comparative cognition research^35^. 1/8 of cashew nut pieces are used as rewards. Details on the device, habituation and training are listed in the ESM section.

### Trial types

We use the following terms throughout the manuscript:

#### Functional trial

A trial where the round button is still rewarding (i.e., before that background’s flashing event).

#### Non-functional trial

A trial where the round button no longer delivers a reward for that specific background, following its flash.

#### Control trial

A trial where both buttons appear on a white background; unlike the coloured-background trials, the round button in control trials never became non-functional.

#### Flash-transition trials

The pair of trials surrounding a flashing event, i.e., the last functional trial immediately before the flash, and the first non-functional trial immediately after the flash.

#### Unequal-status trial

A test trial where the two-coloured/patterned backgrounds currently have different status; one is still functional and the other has already become non-functional.

### Test procedure

During the test phase, the Goffins encountered colourful backgrounds eventually associated with flashing events that rendered the round button non-functional (Video S1; Fig. 1A, B). The experiment was conducted over the course of fifty sessions for each bird, with each session comprising thirty-two test trials and ten control trials (the same with white background; pseudorandomly intermixed with the trials with a colourful background; i.e., control trials were never encountered more than two times in a row). Birds completed a maximum of 2 test sessions per day, with at least 2 h between sessions. In each test trial, a colourful background was presented behind the button on the top half of the screen. The two different backgrounds were presented pseudorandomly (so each background was never encountered more than two consecutive times) in each session. A total of one hundred distinct backgrounds were used throughout the entire test. As mentioned earlier, two colourful backgrounds were used per session so that they could be distinguished from each other. In each session, the two backgrounds appeared 15 times each in a pseudorandom order and intermixed with 10 control trials with a white background. At a randomly selected trial between the 5th and 10th appearance of each background, after pressing the button no food would be released but instead, an event occurred in which the respective background would flash twice in red accompanied with a beeping sound (referred to as the flashing event throughout the text). Thereafter the button of that particular background (not the others) would become non-functional. The only way to exit the slide at this point would be to press the arrow. All backgrounds would reappear as usual but the event would only happen once per background. Nevertheless, when a background reappeared that had already been subjected to the event in a previous trial, its button was still non-functional and the slide would not disappear without pressing the arrow. Each background was associated with a single flashing event per session, but after this event, the round button yielded no reward on every subsequent reappearance of the same background. Thus, although there was only one salient negative event, subjects experienced multiple non-reinforced encounters with that background. The key cognitive contrast is therefore between (a) the first non-functional trial paired with a flash and (b) later non-flash reappearances, which require subjects to identify the background as non-functional without the help of the flash. Ultimately, in the course of a session, both coloured background buttons would eventually become non-functional (but not at the same time) while the white background (control) button would stay intact.

Although we did not independently test colour discrimination in the present experiment, the task did not rely on colour alone, as the backgrounds also differed in pattern. Moreover, parrots possess well-developed colour vision, based on four single-cone classes, and show fine colour discrimination^36^. Thus, the coloured/patterned backgrounds were designed to provide visually discriminable contextual cues.

During the training and testing phase, the experimenter wore mirrored sunglasses and avoided head movements to prevent any potential cues from influencing the birds’ behaviour.

### Data collection

The apparatus automatically recorded the button presses throughout the experiment, with the data ordered by bird identity and session.

### Statistical analysis

All details on model structure (error structure, fixed effects, random effects, random slopes, model reduction, diagnostics, etc.) are provided in the ESM (see section “Statistical analysis in detail”).

Our statistical approach was based on planned analyses of predefined trial categories rather than post hoc subsetting. We did not fit a single model with trial status as one overarching predictor because trial status did not represent a single behavioural contrast across the entire experiment. Functional, non-functional, control, flash-transition, and unequal-status trials differed in what the animal had to do, what constituted a correct response, and, in one case, the scale of the response variable. For the binomial choice models, correctness was defined according to the first button selected in a trial: the round button was coded as correct in functional and control trials, whereas the arrow button was coded as correct in non-functional trials. For the persistence analysis, the response was not binary accuracy but the number of repeated presses on the non-functional round button before switching to the arrow. We therefore analysed each predefined trial category with the model structure appropriate to the behavioural question being tested.

To analyse birds’ performance across different phases of the task, we used a series of Generalized Linear Mixed Models. Logistic GLMMs with binomial error structure were used to model the probability that the first selected button was correct during functional trials (round button; Model A), non-functional trials (arrow button; Model B), control trials (round button; Model C), and the trials immediately before and after a flashing event (round button before the flash; arrow button after the flash; Model D). We further quantified the number of times individuals continued pressing the non-functional round button before switching to the arrow (Model E), and the probability of selecting the correct button when the two backgrounds had unequal states (Model F).

In addition to these main analyses, we conducted within-session background comparison analyses (Models G–I) to examine whether performance differed depending on whether a background was the first or the second to become non-functional within a session. Model G (Poisson GLMM) analysed the number of presses on the non-functional round button after a flashing event. Models H and I (binomial GLMMs) examined the probability of selecting the correct button during the first and second non-functional encounter of each background, respectively.

To gain insight into the overall learning trajectory, we also visualised birds’ performance during the first and the last sessions of the experiment. Furthermore, to assess whether individuals acquired sensitivity to non-functionality, we compared their performance during the first three non-functional trials with the expected performance under the null hypothesis of no learning.

Because the birds were extensively trained to press the round button during functional trials, the appropriate null hypothesis was that they would maintain this response strategy unless they learned that the button had become non-functional. To quantify this, we used the fifth functional trial (the last trial in which both backgrounds were still functional) as the baseline to calculate the expected success rate for non-functional trials under the null hypothesis.

## Supplementary Information

Below is the link to the electronic supplementary material.

Supplementary Material 1

Supplementary Material 2

Supplementary Material 3

## Data Availability

All relevant data are within the manuscript and its Supplementary information files.
