# Supplementary material for: Sensitivity to permanent non-functionality in Goffin’s cockatoos (Cacatua goffiniana)
Source: Sci Rep. 2026 Jun 9;16:26235. doi: 10.1038/s41598-026-57007-1 (PMC13493824; doi:10.1038/s41598-026-57007-1)
Supplement: Supplementary file 1 [file 41598_2026_57007_MOESM1_ESM.docx]

**Sensitivity to permanent non-functionality in Goffin’s cockatoos (*Cacatua goffiniana*)**

Antonio J. Osuna-Mascaró^1^***^†^**, Eleonora Rovegno^1,2^**^†^**, Susana Monsó^3, 4^, Remco Folkertsma^1^, Alice M.I. Auersperg^1^

**SUPPLEMENTARY MATERIAL**

-Video S1
-Supplementary figures (S1, S2, S3, S4, S5, S6 and S7)
-Table S1
-Statistical analysis in detail
-Apparatus details
-Habituation and training procedure
-Supplementary references
-R script and workspace (independent files)

**-Supplementary figures**

Figure S1

Fig S1. Training procedure.
(1) Training phase 1. A single session of forty trials with a white background and a round rewarding button
(2) Training phase 2. Two sessions with twenty pseudorandomized trials consisting of a white background with a round rewarding button and an arrow button to skip trial.
(3) Training phase 3. A single session with thirty trials in which both buttons are present on the same white background, and ten trials presented as in phase 2. Also pseudorandomized.

Figure S2

Fig. S2. Functional trials (Model A).
Probability of choosing correctly during functional trials. The gridded plane represents the group average. Dark bubbles are above the plane, white bubbles are below the plane.

Figure S3

Fig. S3. Controls (Model C).
Probability of choosing correctly during control trials. The color of each line represents the number of non-functional trials present at the moment. Due to the pseudorandomness of controls, the size of the bubbles represents the number of interactions that they had with each.

Figure S4

Fig. S4. Performance while being wrong (Model E).
Number of round button-presses during non-functional trials, across trials and sessions. the gridded plane represents the group average. Dark bubbles are above the plane, white bubbles are below it.

Figure S5

Fig. S5. Unequal condition (Model F).
Probability of choosing correctly during unequal conditions. Color represents when facing functional (blue) and non-functional (pink) trials.

Figure S6

**1**

**2**

Figure S6. Probability of choosing correct button during the first and second encounter of a non-functional screen.
Lines represent model estimates and 95% confidence intervals. Points depict from raw data per session calculated average percentage correct choices. Performance comparison between the first and second background to become non-functional within a session. Panel 1 shows performance during the first encounter; Panel 2 shows performance during the second encounter. Here, A and B refer to the two coloured/patterned backgrounds presented within a given session.

Figure S7

Fig. S7. First and last five sessions for each subject.
Average performance when close to the change in functionality along the whole experiment and a comparison between the first and the last five sessions for each individual. Red column represents the change in functionality, all trials at the left are functional and all trials at the right are non-functional. The performance for both backgrounds is compressed here. Color black represents the average performance for the whole experiment, orange represents the average for the first five sessions, and red represents the average for the last five.
(A) Highlights two individuals, Doree (DOR) with excellent performance on the first and second non-functional trials during the final sessions, and Renki (REN) with a good performance but a drop during the first and the second non-functional trials.
(B) Individualized performance of all subjects.

-**Table S1**

| Distance to change in functionality \| Non-functional trial.nr | Sessions | Percentage correct | lowerCI | upperCI | Z-score | P-value H_0_ [last non-functional trial overall]: 1-.915 = .085 |
| --- | --- | --- | --- | --- | --- | --- |
| 1 | 1-5 | 0.286 | 0.1780 | 0.426 | 4.653735 | 0.0000 |
| 1 | 46-50 | 0.807 | 0.6800 | 0.891 | 11.06475 | 0.0000 |
| 2 | 1-5 | 0.336 | 0.2350 | 0.454 | 6.6403 | 3.13E-11 |
| 2 | 46-50 | 0.431 | 0.2770 | 0.599 | 6.0238 | 1.70E-09 |
| 3 | 1-5 | 0.414 | 0.2730 | 0.571 | 6.2795 | 3.40E-10 |
| 3 | 46-50 | 0.518 | 0.2960 | 0.733 | 5.1125 | 3.18E-07 |

Table S1. Performance during the first, second, and third non-functional trials at the beginning (sessions 1 to 5) and at the end of the experiment (sessions 46 to 50) relative to the null hypothesis (the opposite of their overall performance during the fifth trial).

**-Statistical analysis in detail**

Across experiment: functional, non-functional, and control trials.

In order to model the probability of correct choices over the entire course of the experiment we fitted three generalized linear mixed models (GLMM^1^). In each of these models the response was pressing the correct button in a trial, and thus we ran logistic regression with binomial error structure and logit link function. Each of these models differed in the data used. For Model A we only included functional trials, for Model B we only included non-functional trials, and for Model C we only included control trials.

Model A and Model B were similar in structure. As key fixed effect predictors we included session number, trial number and their interaction. As control fixed effect predictors, we included sex and status of the other background included in the session. We included a number of random intercept effects. We included subject to avoid pseudoreplication and account for repeated measurements of the same individual. To model possible within individual session to session variation we included session nested within individual sessionID as random intercept effect. Finally, to model background associated variation, we included backgroundID as random intercept effect. To keep the type I error rate at the nominal level of 0.05^2,3^ we included random slopes of session number, trial number and their interaction and status of the other background in subject; random slopes of trial number and status of the other background in sessionID; and random slopes of session number, trial number and their interaction, status of the other background and sex in backgroundID.

Model C included as key fixed effect predictors session number, trial number, the number of non-functional screens, and all their interactions up to the third order. We included the three-way interaction as we expected that the birds’ performance would change with experience and that this might be differently influenced by the number of non-functional screens (e.g. over time birds might get less distracted by the presence of non-functional screens as in the beginning). As control fixed effect predictor we included sex. As random intercept effects, we included subject to avoid pseudoreplication and account for repeated measurements of the same individual and sessionID to model possible within individual session to session variation. To keep the type I error rate at the nominal level of 0.05^2,3^ we included random slopes of the three-way interaction between session number, trial number, the number of non-functional screens in subject; and random slopes of trial number and the number of non-functional screens in sessionID.

Change event trials

In order to model the probability of correct choice during the change event trials we fitted a GLMM with binomial error structure and logit link function (Model D). This model only included data from trials adjacent to the change in functionality, that is, the last trial before the change of functionality and the first trial after the change of functionality (2 trials for each of the backgrounds in a session). We included pressing the correct button as a response, which is round in the trial before the change and arrow in the trial after the change. In the model, we included session number, background status and their interaction as key fixed effect predictors. As control fixed effect predictors we included sex and status other background. We used a similar random intercepts effects structure as Model A. We included subject to avoid pseudoreplication and account for repeated measurements of the same individual. To model possible within individual session to session variation we included session nested within individual sessionID as random intercept effect. Finally, to model background associated variation, backgroundID was included as random intercept effect. To keep the type I error rate at the nominal level of 0.05^2,3^ we included random slopes of the interaction between session number and background status, and status of the other background in subject; random slopes of the interaction between session number and background status, status of the other background and sex in backgroundID; and random slopes of background status and status of the other background in sessionID.

Unequal-status trials

In order to model the probability of correct choices during the unequal-status trials we fitted a GLMM with binomial error structure and logit link function (Model F). This model only included data from test trials in which the backgrounds are of different status, that is, when one background has gone non-functional, while the other background is still functional. We included pressing the correct button as a response, which is different for each of the backgrounds, the round button for the still functional background, and arrow button for the non-functional background. In the model, we included session number, background status and their interaction as key fixed effect predictors. Sex was included as control fixed effect predictor. We used a similar random intercepts structure as model A. As random intercept effect we included subject to avoid pseudoreplication and account for repeated measurements of the same individual. To model possible within individual session to session variation we included session nested within individual sessionID as random intercept effect. And, to model background associated variation, backgroundID was included as random intercept effect. To keep the type I error rate at the nominal level of 0.05,^2,3^ we included random slopes of the interaction between session number and background status in subject; random slopes of session number, background status, and sex in backgroundID; and the random slopes of background status in sessionID.

Pressing the incorrect button.

To model the extent to which birds keep on pressing the non-functional button after a background turned non-functional we fitted a GLMM with Poisson error structure and log link function. This Model E only included only data from non-functional trials. We included the number of times the round button was pressed in each trial (range: 0-34 times) as a response. As key fixed effect predictors we included session number, trial number and their interaction, as control fixed effect predictors we included sex and the status of the other background. We used a similar random intercepts structure as Model A. As random intercept effect we included subject to avoid pseudoreplication and account for repeated measurements of the same individual. To model possible within individual session to session variation we included session nested within individual sessionID as random intercept effect. And, to model background associated variation, backgroundID was included as random intercept effect. To keep the type I error rate at the nominal level of 0.05,^2,3^ we included random slopes of the interaction between session number and trial number and status of the other background in subject; random slopes of the interaction between session number and trial number, status of the other background, and sex in backgroundID; and the random slopes of trial number in sessionID.

Beyond the task-level analyses described above, we also examined within-session variation to test whether birds performed differently depending on whether a background was the first or second to become non-functional.

Within-session background comparison analyses (Models G–I)

To investigate whether birds’ performance differed depending on whether a background was the first or second to become non-functional within a session, we fitted three additional mixed-effects models.

Model G: Incorrect button presses after a background becomes non-functional

We fitted a GLMM with Poisson error structure and log link function. This model included only trials in which the round button was already non-functional. The response variable was the number of times the round button was pressed within a trial after the flashing event (range 0–34). As key fixed-effect predictors, we included session number, trial number, background number (first or second background to become non-functional), and all interactions up to the three-way interaction. Sex and the status of the other background were included as control predictors.

Random intercept effects were included for subject, sessionID nested within subject, and backgroundID. To keep the type I error rate at the nominal level of 0.05, we included random slopes of the three-way interaction and the status of the other background in subject; random slopes of the interaction between session number and trial number, sex, status of the other background, and background number in backgroundID; and the random slopes of the interaction between trial number and background number in sessionID.

Model H: Correct button choice during the first non-functional encounter

This model examined whether the probability of selecting the correct button during the first non-functional encounter differed between backgrounds. We fitted a binomial GLMM with logit link, with “correct button press” as the response. Key fixed-effect predictors were background number, session number, and their interaction. Sex was included as a control predictor.

Random intercept effects were included for subject, sessionID, and backgroundID. To keep the type I error rate at the nominal level, we included random slopes of the interaction between background number and session number within subject; and random slopes of session number and sex within backgroundID.

Model I: Correct button choice during the second non-functional encounter

Using the same model structure as Model H, we analysed behaviour during the second non-functional encounter of each background. Fixed and random effects were identical to Model H, and correct button press was again the response. This model tested whether birds’ ability to identify the non-functional background differed between the first and second background to become non-functional during the second non-functional encounter.

General considerations.

All models were fitted in R (version 4.2.3^4^), using the glmer function of the ‘lme4’ package (version 1.1-27.1^5^), with the optimizer ‘bobyqa’.^6^ Before being included in the model session number and trial number were z-transformed to a mean of zero and standard deviation of one to ease model convergence.^7^ Random slopes of factors (status other background and sex) were dummy coded and centered before being included as random slopes. Random slopes were included only when the corresponding predictor varied within the levels of the relevant grouping factor. Sex was therefore not included as a random slope for subject or sessionID, because each subject, and consequently each subject-specific session, had only one sex. Where applicable, sex was included as a random slope for backgroundID because backgrounds were encountered by individuals of both sexes. In the random effects part of each model we started by including the correlation between random intercepts and random slopes, but we removed them when they appeared in part unidentifiable (indicated as being close to +1 or -1) to balance type I and type II error rates.^8^

After fitting each model, we checked for violations of model assumptions and assessed model stability. For each model we visually confirmed that the best linear unbiased predictors (BLUPs) per level of the random effects were approximately normally distributed. We verified the absence of collinearity by calculating the Variance Inflation Factor (VIF) for the model excluding any interactions and random effects using the package ‘car’ (version 3.0-12^9^). This revealed that collinearity was not an issue in any model (max VIF = 1.20). For each model we assessed model stability by excluding levels of random intercept effects one at a time and comparing the resulting estimates with those obtained from the model based on all data,^10^ we determined that all models were of good stability. We checked for overdispersion in model 3, with a dispersion parameter of 1.02 the model was not overdispersed.

For each model we first tested the overall effect of key fixed effect predictors to avoid an increased type I error risk due to cryptic multiple testing,^11^ we did this by comparing each full model to its respective null model lacking key predictors but being otherwise identical using a likelihood ratio test.^12^ If this revealed significance we continued with testing individual fixed effects by dropping them from the model one at a time and comparing the simpler with the more complex model and utilizing likelihood ratio tests by using the drop1 command. We started with testing the highest order interactions and removing non-significant interactions until none of the non-significant interactions were left to be able to inspect the significance of lower order terms. We used the function bootMer of the lme4 package to determine confidence intervals of model estimates by means of parametric bootstrapping (N = 1,000 bootstraps). Figures were created using the ‘ggplot2’ package.^13^

Performance after flashing event

In order to investigate how individuals responded to the flashing event, we compared the performance during non-functional trials 1, 2, and 3 during the first five sessions (session 1 to 5) and last five sessions (session 46 to 50) to the birds’ expected performance as if they had not acquired sensitivity to non-functionality. For this we subtracted their performance in the last trial during which no non-functional screens is presented (i.e., trial number -6) from 1. For this we built six separate logistic regressions, one for each non-functional trial and session set combination. In each of these we used correct button as the response variable, with session number (z-transformed), sex and status other background as fixed effects; and subject (including the random slope of session number and status other background), backgroundID and sessionID as random intercept effects. The intercept in each of these models thus represents the sample mean performance as estimated by the model. The intercepts p-value indicates whether the intercept deviates significantly from 0 (i.e., the null-hypothesis for the intercept is intercept==0), which in a logistic model represents a probability at chance level (P=0.5). Our null-hypothesis is the probability of being correct at P=0.085 (final trials performance). We therefore tested for a significant deviation from the expected performance level by subtracting the logit-transformed chance-value of 0.085 from each model estimate and calculated the corresponding z-value and p-value of these adjusted estimates. Thus, by testing the significance of the intercept of these models we tested if individuals overall differed from their expected performance without acquired sensitivity to non-functionality.

**-Apparatus details**

The device is designed for easy accessibility, with a keyboard located on the right side of the box, which can be elevated to reveal the internal CPU. The CPU consists of a Mini-ITX mainboard (VIA EPIA1 M10000) with a 1-GHz processor. It features two USB ports, one LAN port (10/100 Mbit), integrated sound, VGA, 512 MB DDR RAM, and a 40-GB 2.5-inch hard drive. The frontal side of the apparatus has a height of 75 cm and a width of 50 cm. The capacitive touchscreen (30 × 23 cm) is located on the top half of the front side of the apparatus. A wooden cylindrical perch, 40 cm in length and elevated 23 cm above the ground, is situated slightly separated from the screen (see Fig. 1). The screen itself is XGA color TFT LCD module (Model G150XG01 by AU Optronics Corp., Taiwan; http://www.auo.com), offering a resolution of 1024 × 768 px. An IR “CarrollTouch” touch frame (Model D87587-001, without filter) by Elo (Menlo Park, CA; http://www.elotouch.com) is attached to the screen to detect responses. The reward delivery opening is centrally positioned, 80 mm below the lower edge of the screen, allowing subjects comfortable interaction. Below the perch, the apparatus contains a small container to catch the reward falling out of the apparatus. Small pieces of cashew nuts are used as rewards. The rewards will fall every time that a functional round button is pressed first during a trial.

**-Habituation and training procedure**

**Habituation phase**

The first step in preparing the Goffins for the study was the familiarization phase, which consisted of several incremental steps. During the initial group habituation, additional colony members could be present in the testing area, but only the fifteen birds included in the final sample proceeded through the full training and test procedure. The subjects were introduced to the touch screen apparatus screen inside the testing chamber, first as a group and later either individually or in small groups of four to five birds. Those birds that were more reluctant to approach the device were habituated in these small groups in order to use social facilitation to overcome neophobia.

During habituation, the touchscreen was turned on with the screen completely white. The flashing event was accompanied by an auditory beep, as shown in Video S1. Food rewards such as unshelled sunflower seeds and drops of baby formula for parrots (Nutribird) were used to motivate the birds to approach the apparatus. Habituation was considered complete when a bird was readily sitting on the perch and confidently touching the screen.

**Training, phase 1**

In the initial phase of the three-step training process, the birds were conditioned to respond to a circular reward-prompting button (see Fig. S1). This was achieved through a series of 40 trials, during which the screen displayed only a circular black button against a white background. Upon beak contact with the button, it would disappear for a period of three seconds. During this time, a small quantity of cashew was dispensed into the container located beneath the perch. Once the three-second interval had elapsed, the button reappeared, initiating a new trial.

**Training, phase 2**

The second step of training was designed to familiarize the birds with the function of the arrow button, which would allow them to skip the trial. This second phase of the training involved two sessions of forty trials. Twenty trials presented the round button in the same manner as in the previous phase, and the rest featured only an arrow button alone in the lower half of the screen. In contrast to the round button, the arrow button was not associated with a reward. The trials were pseudorandomized in such a way that no more than two consecutive similar trials were permitted during a session.

**Training, phase 3**

The third and final training step was designed to confirm that the birds had learned to operate both buttons. As before, when a subject presses the arrow button to skip a trial, a distinctive single beep is heard and both buttons disappear. Three seconds later, the buttons reappear for a different trial. In this training phase both buttons were present in the same slide (top and bottom half). We tested whether the birds showed a preference for the round button in this case, pressing the arrow button only when it is presented alone. This final phase of the training procedure involved a single session of forty trials. In thirty trials slides included both buttons, while in ten trials slide incorporated only the arrow button. The order of trials was pseudorandomized as in phase 2.

**-Supplementary references**

1.	Baayen, R. H. *Analyzing Linguistic Data: A Practical Introduction to Statistics Using R*. (Cambridge University Press, Cambridge, 2008). doi:10.1017/CBO9780511801686.

2.	Barr, D. J., Levy, R., Scheepers, C. & Tily, H. J. Random effects structure for confirmatory hypothesis testing: Keep it maximal. *Journal of Memory and Language* **68**, 255–278 (2013).

3.	Schielzeth, H. & Forstmeier, W. Conclusions beyond support: overconfident estimates in mixed models. *Behav Ecol* **20**, 416–420 (2009).

4.	R Core Team. R: A language and environment for statistical computing. R Foundation for Statistical Computing, Vienna, Austria. https://www.eea.europa.eu/data-and-maps/indicators/oxygen-consuming-substances-in-rivers/r-development-core-team-2006 (2022).

5.	Bates, D., Mächler, M., Bolker, B. & Walker, S. Fitting Linear Mixed-Effects Models Using lme4. *Journal of Statistical Software* **67**, 1–48 (2015).

6.	The BOBYQA algorithm for bound constrained optimization without derivatives. Preprint at https://optimization-online.org/?p=11137 (2010).

7.	Schielzeth, H. Simple means to improve the interpretability of regression coefficients. *Methods in Ecology and Evolution* **1**, 103–113 (2010).

8.	Matuschek, H., Kliegl, R., Vasishth, S., Baayen, H. & Bates, D. Balancing Type I error and power in linear mixed models. *Journal of Memory and Language* **94**, 305–315 (2017).

9.	Fox J, Weisberg S. *An R Companion to Applied Regression.* (Sage, Thousand Oaks, CA, 2019).

10.	Nieuwenhuis, R., Grotenhuis, M. te & Pelzer, B. influence.ME: tools for detecting influential data in mixed effects models. *R journal* **4**, 38–47 (2012).

11.	Forstmeier, W. & Schielzeth, H. Cryptic multiple hypotheses testing in linear models: overestimated effect sizes and the winner’s curse. *Behav Ecol Sociobiol* **65**, 47–55 (2011).

12.	Dobson, A. J. & Barnett, A. G. *An Introduction to Generalized Linear Models*. (Chapman and Hall/CRC, New York, 2018). doi:10.1201/9781315182780.

13.	ggplot2: Elegant Graphics for Data Analysis (2nd ed.): Measurement: Interdisciplinary Research and Perspectives: Vol 17 , No 3 - Get Access. https://www.tandfonline.com/doi/full/10.1080/15366367.2019.1565254.
